# Comprehension of polarity of articles by citation sentiment analysis using TF-IDF and ML classifiers

**DOI:** 10.7717/peerj-cs.1107

**Published:** 2022-12-13

**Authors:** Musarat Karim, Malik Muhammad Saad Missen, Muhammad Umer, Alisha Fida, Ala’ Abdulmajid Eshmawi, Abdullah Mohamed, Imran Ashraf

**Affiliations:** 1 Department of Computer Science & Information Technology, Islamia University, Bahawalpur, Bahawalpur, Pakistan; 2 University of Jeddah, Department of Cybersecurity, College of Computer Science and Engineering, Jeddah, Saudi Arabia; 3 University Research Centre, Future University in Egypt, Cairo, Egypt; 4 Information and Communication Engineering, Yeungnam University, Gyeongsan, Korea

**Keywords:** Citation sentiment analysis, SMOTE, Machine learning, Term frequency-inverse document frequency, Dataset balancing

## Abstract

Sentiment analysis has been researched extensively during the last few years, however, the sentiment analysis of citations in a research article is an unexplored research area. Sentiment analysis of citations can provide new applications in bibliometrics and provide insights for a better understanding of scientific knowledge. Citation count, as it is used today to measure the quality of a paper, does not portray the quality of a scientific article, as the article may be cited to indicate its weakness. So determining the polarity of a citation is an important task to quantify the quality of the cited article and ascertain its impact and ranking. This article presents an approach to determine the polarity of the cited article using term frequency-inverse document frequency and machine learning classifiers. To analyze the influence of an imbalanced dataset, several experiments are performed with and without the synthetic minority oversampling technique (SMOTE) and uni-gram and bi-gram term frequency-inverse document frequency (TF-IDF). Results indicate that the proposed methodology achieves high accuracy of 99.0% with the extra tree classifier when trained on SMOTE oversampled dataset and bi-gram features.

## Introduction

Sentiment analysis has been studied extensively during the last few years due to the inception and evolution of microblogging websites and social media platforms like Twitter, Instagram, Facebook, etc. The analysis of sentiments expressed in tweets, reviews, and comments helps public and private sector companies and organizations to introduce and improve the products for customers’ higher satisfaction. Besides, it facilitates the policymakers, government officials and politicians in understanding public sentiments about particular policies, services and popularity of individuals (Prabowo & Thelwall, 2009). Citation sentiment analysis is a relatively new approach that focuses on determining the sentiment of a cited scientific article.

For the evolution of scientific research, sometimes novel ideas emerge, however, predominantly, the old ideas are improved to overcome their limitations. For conducting experiments, the researcher uses previous publications as the baseline on which their improved models are built. Two approaches are adopted by the researchers: use the old approach for their problem and criticize the approach by highlighting its limitations. The former approach is an example of positive citation while the latter represents the negative citation (Liu, 2017). A neutral citation is when a previous work is stated as an example of the similarity between your work and the previous work. We can say that the positive, negative, and neutral citations are used for acknowledging, criticizing, and comparing the previous work with the current work (Hernández & Gómez, 2014).

Citation analysis is an important task in characterizing the importance and scope of scientific knowledge. For example, the number of citations of a research article is used to determine the impact of an article (Yousif et al., 2019). An article that gets a higher number of citations is entitled to higher impact or quality. However, this evaluation method is both inappropriate and misleading as it does not consider the sentiment of a citation (Abu-Jbara, Ezra & Radev, 2013). An article may be cited negatively to discuss its limitations, as well as, neutrally for performance comparison. For this reason, the citation count does not necessarily portray the quality of a research article. Consequently, several researchers focused on analyzing the sentiment of a citation to quantify the quality of the cited article.

Citation sentiment is determined using the sentiment analysis techniques used for the opinionated text from social media platforms where its sentiment is determined to be positive, negative, or neutral. A similar concept is used for citation sentiment where the context of the citation is analyzed by analyzing the reason for the citation (Teufel, Siddharthan & Tidhar, 2006). Sentiment citation could provide valuable insight into a particular research article and depict the research gap. For example, a positive citation indicates the strength of its proposed approach and quality. A citation is considered positive if the cited article is used for comparison and shows good performance (Abu-Jbara, Ezra & Radev, 2013). If the comparison shows poor results, the citation is negative which indicates the weakness and limitations of the cited article. Neutral citation is used for description only and does not state the advantages or disadvantages of the article, as articles make citations for the description of different methods and algorithms.

Analyzing the sentiments/purpose of citation from the literature is a laborious and time-consuming process. As a result, several automatic sentiment analysis approaches have been proposed (Abu-Jbara, Ezra & Radev, 2013; Jochim & Schütze, 2014; Athar, 2011). However, such approaches are complex and require several features for determining the citation sentiment. Moreover, large datasets with annotations are required to achieve better accuracy. This research proposes a methodology that achieves higher accuracy than that of existing state-of-the-art approaches with a smaller dataset. The main contributions of this study can be summarized as follows:

• A novel methodology is proposed to determine the sentiment of a citation into positive, negative, and neutral. It does not require a large dataset to achieve high accuracy. For feature extraction, term frequency-inverse document frequency (TF-IDF) uni-gram and bi-gram features are used.
• Several machine learning algorithms are tested with the proposed methodology including decision tree (DT), AdaBoost classifier (AB), logistic regression (LR), stochastic gradient classifier (SG), random forest (RF), extra tree classifier (ET), support vector classifier (SV) and a voting classifier (VC) which combines LR and SG.
• The influence of dataset imbalance on the sentiment classification is analyzed through several experiments. Similarly, the impact of the down-sampled balanced dataset is evaluated with the selected classifiers. Additionally, the synthetic minority upsampling technique (SMOTE) is utilized to balance the dataset and its efficacy is investigated.

## Related Work

Several measures have been presented over the years to determine the quantitative quality or importance of an author or author’s work. For example, the h-index is an important measure to determine the importance or rank of an author (Hirsch, 2005). Similarly, author eigenvector and author impact factor are other important measures to determine the quality (West et al., 2013; Pan & Fortunato, 2014). However, qualitative measures to estimate the rank of an article are not explored properly. Citation sentiment is relatively new but interesting research is to overcome the limitations of quantitative measures for analyzing the importance of scientific articles and many approaches can be found in the literature for citation sentiment.

The authors present a hybrid model in Kaur & Ojha (2020) where the objective and subjective measures are combined to assess the impact of a research article. For this purpose, the publication’s impact factor and author’s impact factor are combined with the citation sentiment analysis to determine the paper’s impact. Citation sentiment analysis is carried out using the SentiWordNet 3.0 in a stand-alone environment. The sentiment score is calculated between 0.0 to 1.0 for positive while below zero for negative sentiment. For calculating the citation sentiment, a complex process is followed which uses tokenization and lemmatization as initial steps. Later tagging is done for each lemma and its score is calculated using the SentiWordNet. Scores varying between 0.0 and 0.4 show a neutral sentiment.

Besides using the citation sentiment with objective measures, several approaches focus only on the citation sentiment and adopt various models to do that. For example, the study (Ikram & Afzal, 2019) proposes a two-level citation sentiment analysis approach by identifying the aspect-level sentiments. Initially, various aspects are extracted from the citation sentences with the help of text surrounding the citation. These aspects are later used to determine sentiment polarity using a linguistic rule-based approach. N-gram features are utilized for the proposed approach with a support vector machine to achieve high citation sentiment classification accuracy. Similarly, the authors present a deep learning approach for article sentiment analysis in Nguyen et al. (2017). An LSTM approach is adopted in the proposed methodology with several dropout layers to prevent overfitting. Word dimensionality is reduced using the word embedding model with word2vec features. The imbalance data for positive, negative, and neutral classes is dealt with SMOTE sampling approach. Results indicate the better performance of the proposed approach as compared to the traditional SVM algorithm.

A context-based citation sentiment classification is done in Athar & Teufel (2012). A new sentiment corpus is presented which is annotated with the dominant sentiment. Experiments are performed to analyze the impact of context window size on various approaches. Results using N-gram features show that the introduction of contexts increases the vocabulary size and affects performance. The importance of considering citation sentiment for ranking a paper is addressed in Ghosh & Shah (2020) where citation sentiment is performed on ACL paper collection. Several classifiers from WEKA are trained using the selected features such as sentiment score, n-grams with positive and negative polarity, part-of-speech tags, self-citation, and sentiment words. Experiment results show that Dagging, a meta-classifier, proves to be the best performer with an accuracy of 80.61%.

The authors present a citation strength estimation approach in Wan & Liu (2014) to explain that all the citations in a research paper are not equally important and simple counting of the citations is not an appropriate approach to determine their importance and strength. The problem is taken as a regression task and *ϵ*-SVM is applied to address the problem. Several important features are selected to determine the strength of the citation such as occurrence number, located section, time interval, the average length of citing sentence, the average density of citation occurrences, and self-citation. Results suggest that the proposed approach can achieve results that are very similar to the human evaluator. Another study that uses the citation sentiment to determine the quality of an article is Sendhilkumar, Elakkiya & Mahalakshmi (2013) where the quality of an article is estimated using its citation quality. Three semantic related characteristics are used to find the citation quality including citation classification, citation sentiment analysis, and content relevancy. Citation sentiment analysis is performed using the SentiWordNet with citation context using the part-of-speech tag. Results of the supervised machine learning approach with CRFs show that the proposed approach can estimate the strength of an article using the citation strength and parse the citation into eight fields.

In addition to using citation sentiment with other factors to determine a research article’s strength and quality, several approaches focus mainly on estimating the sentiment of a citation. For example, the authors present a machine learning approach using the word2vec features for citation sentiment in Liu (2017). The sentence vectors are constructed using the word embeddings from ACL collections. The negative and positive polarity of an embedding is utilized to determine the polarity of the citation. The selected features are used with SVM to estimate the sentiment of a citation. Results with 10-fold cross-validation prove that handcrafted features show better performance to determine the polarity of a citation. In the same vein, a deep learning approach, called ImpactCite, is proposed in Mercier et al. (2020). The proposed approach is based on XLNet which focuses on the tasks of sentiment classification and intent classification where intent citation shows the purpose of the paper where the citation is made. Experiments are performed with CNN, LSTM, and RNN networks and SMOTE sampling approach. Impact Cite shows scores of 88.13 and 88.93 for micro-f1 and macro-1, respectively, and outperformed existing citation sentiment approaches.

Despite the accuracy reported in the above-cited research works, they have several limitations. First, the process followed to determine the sentiment of a citation in many approaches is very complex involving part-of-speech tags, and manual feature extraction and labeling. Secondly, the reported accuracy is still low to determine the quality and importance of a scientific article and requires more robust and accurate models for the task. Thirdly, acquiring a higher accuracy with the existing approaches requires large annotated datasets. Lastly, many of the discussed approaches use word2vec features, and other features like TF-IDF with various grams and global vectors (GloVe), *etc*., are not well studied. Similarly, a few machine learning algorithms like SVM are investigated and many important classifiers like LR, and ET, are not used. This study aims to leverage TF-IDF features with various machine learning algorithms to enhance the accuracy of citation sentiment.

## Materials and Methodology

This section provides the details for the dataset used for experiments, machine learning algorithms, proposed methodology, and the accuracy measures used to evaluate the performance of the proposed methodology.

### Dataset used for experiments

This study uses the ‘citation-sentiment-corpus’ provided by Athar (2011). The corpus contains 8,736 citation sentences which are manually annotated using the sentences from various papers. The citation sentences which are used to annotate the sentiments are taken from the ACL anthology network corpus. The dataset comprises four attributes and names and the meaning of each attribute is given in Table 1.

The annotations are labeled manually using the sentiments found in the ‘Citation_Text’. The value of the sentiment is determined based on the intent of the citing author, *e.g.*, criticism, acknowledgment, comparison, *etc.*, The value of the sentiment can be ‘p’, ‘n’, and ‘o’ for positive, negative, and objective, respectively. Sample records for each sentiment from the datasets are shown in Table 2.

### Machine learning classifiers used for experiments

For the current study, several machine learning algorithms are used for their citation sentiment classification accuracy. The selected machine learning algorithms include DT, AB, LR, SG, RF, GB, ET, NB, and SVM.

#### Decision tree

Decision trees are one of the most widely used machine learning algorithms for classification problems. DT performs well both on the categorical, as well as, numerical data (Breiman et al., 1984). For understanding features and inferring decisions, DT is a simple but powerful tool. To infer decisions, DT follows a tree-like structure which is composed of three kinds of nodes: root node, internal node, and leaf node (Tan, Steinbach & Kumar, 2006). The root node has no incoming edges and zero or more outgoing edges, the internal node has one incoming and two or more outgoing edges and the leaf node has one incoming edge only (Ashraf, Hur & Park, 2018). To determine the goodness of fit, the gain ratio is used as the split criteria

(1) \begin{eqnarray*}Gain~ratio= \frac{{{null}}_{info}}{Split~Info} \end{eqnarray*}

where,

(2) \begin{eqnarray*}Split~Info=-\sum _{i=1}^{k}P({v}_{i})lo{g}_{2}P({v}_{i})\end{eqnarray*}

**Table 1:** Names and description of dataset attributes.

Attribute	Description
Source_Paper_ID	Paper ID from where the cited text is taken
Target_Paper_ID	Paper ID which is cited
Sentiment	Sentiment of the citation
Citation_Text	Actual text taken from source paper

**Table 2:** Sample records from the dataset.

Source_Paper_ID	Target_Paper_ID	Sentiment	Citation_Text
E06-1040	P02-1040	p	“Some NLG researchers are impressed by the success of the BLEU evaluation metric (Papineni et al., 2002) in Machine Translation (MT), which has transformed the MT field by allowing researchers to quickly and cheaply evaluate the impact of new ideas, algorithms, and data sets.”
W06-2207	P95-1026	n	“Although a rich literature covers bootstrapping methods applied to natural language problems (Yarowsky, 1995; Riloff, 1996; Collins and Singer, 1999; Yangarber et al., 2000; Yangarber, 2003; Abney, 2004) several questions remain unanswered when these methods are applied to syntactic or semantic pattern acquisition.”
I05-2009	A00-2024	o	“5.3 Related works and discussion Our two-step model essentially belongs to the same category as the works of (Mani et al., 1999) and (Jing and McKeown, 2000).”

where *k* shows the number of splits. DT is favorable due to being non-parametric, computationally inexpensive, and easy to interpret.

#### AdaBoost classifier

AdaBoost is a boosting classifier that works with several weak learners into strong learners (Freund, Schapire & Abe, 1999). AB maintains a *W* weight distribution for a given set of training samples. This distribution *W*_*t*_ is updated with each cycle concerning the output results and weights are assigned as low and high for easy and hard samples, respectively (Schapire & Singer, 1999). Following this process, AB focuses on hard samples and within given *T* cycles, it combines the component classifiers into a single final hypothesis. AB has a strong property where the training error of the final hypothesis drops exponentially if the component classifiers have slightly better accuracy (Li, Wang & Sung, 2008).

#### Logistic regression

Predominantly, LR is used for the classification problems (Boyd, Tolson & Copes, 1987). It is well suited for problems where the relationship between a categorical variable and one or more categorical predictors is to be determined (Peng, Lee & Ingersoll, 2002). LR solves the problems of non-linearity, and non-uniform distribution using the logit transformation. A simple logistic model can be represented as

(3) \begin{eqnarray*}logit(Y)=natural~log(odds)=ln \left( \frac{\pi }{1-pi} \right) =\alpha +\beta X.\end{eqnarray*}

#### Stochastic gradient descent

Stochastic gradient descent is a popular algorithm used for various machine learning tasks. SG combines several binary classifiers to form a one-versus-all method (Gardner, 1984). It follows an iterative process starting from a random point and travels through the slope to reach the lowest point. The working mechanism follows a regression approach and is easy to implement and interpret. SG is preferred for the large dataset as it considers all the training samples in each iteration. To obtain high classification accuracy, several hyperparameters of SG are evaluated and their optimal values are set. For feature scaling, the SG has high sensitivity.

#### Random forest

Random forest is an ensemble model that follows a tree-based approach (Breiman, 2001). Each tree in the RF is generated with a random vector and holds a unit vote for the input vector (Deng et al., 2008). RF has attribute selection and pruning measures and gains ratio and Gini index are mostly used for attribute selection (Mitchell, Michalski & Carbonell, 2013). RF is preferred for its capability to handle sparse datasets, contain errors, or have missing values. Several hyperparameters can be used to enhance its performance including the number of features, number of trees, maximum depth, confidence level, gain, leaf size, and number of pre-pruning alternatives.

#### Gradient boosting

The gradient boosting technique is used for regression and classification problems and it follows a TEE-based approach. GB combines many weak classifiers to create a strong learning model (Friedman, 2001). It has shown good performance in many practical applications of machine learning (Johnson & Zhang, 2013). The strength of the learning model is improved using several weak learners through the process called probability approximately correct learning. It leads to show good performance on the unprocessed data where the data has missing values. Various loss functions can be used with the GB, and the gradient descent method is the common selection

(4) \begin{eqnarray*}{y}_{i}^{p}={y}_{i}^{p}-\alpha \ast \sum \left( {y}_{i}-{y}_{i}^{p} \right) \end{eqnarray*}

where *α* and $\sum \left( {y}_{i}-{y}_{i}^{p} \right) $ represents the learning rate and sum of residuals, respectively.

#### Extra tree classifier

An extra tree classifier is an ensemble approach that follows a similar working mechanism to that of a random forest and aggregates the results of multiple decision trees. However, it uses a different method for constructing the trees in the forest (Sharaff & Gupta, 2019). Unlike the RF which subsamples the input data, the ET utilizes the whole input data and does not use bootstrap replicas by default. Another striking difference is the selection of cut points where ET chooses the cut points randomly in comparison to the RF which chooses the optimal split. Random samples of *k* best feature are taken to infer decisions and the Gini index is used for feature selection to split the data in a tree. Consequently, ET reduces both the bias and variance and computational cost is low due to its random selection criteria.

#### Gaussian naive bayes

Gaussian Naive Bayes is a variant of Naive Bayes that uses the Gaussian distributions and continuous data (Perez, Larranaga & Inza, 2006). It uses the prior and posterior probability of the classes in the data. The posterior probability *P*(*H*|*X*) is calculated for given data samples *X* = {*x*_1_, *x*_2_, …, *x*_3_} with *n* attributes and *C* class labels. Bayes rule determines *P*(*C*|*X*) as

(5) \begin{eqnarray*}P({C}_{i}{|}X)= \frac{P(X{|}{C}_{i})P({C}_{i})}{P(X)} .\end{eqnarray*}

Despite being simple, NB often outperforms many sophisticated classification models (Twala, 2010). The gaussian naive Bayes uses the features that have continuous values and assumes that those features follow a Gaussian or normal distribution.

### Support vector machines

Originally developed by Cortes & Vapnik (1995) Support Vector Machine is a supervised learning technique that is widely used for non-linear classification, regression, and outlier detection (Schölkopf, Burges & Vapnik, 1996; Bennett & Campbell, 2000). The classification or class separation uses high-dimensional hyperplanes which are drawn to maximize the distance between the samples of different classes. The points that lay on the boundaries are known as support vectors. These hyperplanes are determined using the quadratic programming optimization problem and the distance between the planes determines the distinctiveness of the classes (Shmilovici, 2009).

Various hyperparameters for each machine learning classifier are fine-tuned to achieve a higher citation sentiment accuracy. A list of all the parameters and their associates that are used in the current study is given in Table 3. The dataset is split with a 70:30 ratio for training and testing, respectively using the following method.

**Table 3:** Hyperparameters and their associated values which are used for experiments.

Classifier	Values for hyperparameter
RF	n_estimators=100, random_state=52
AB	n_estimators=100, random_state=52
DT	random_state=50,
SV	kernel=‘linear’, *C* = 2.0, random_state=52
ET	n_estimators=100, random_state=52
GB	max_depth=100, learning_rate=0.1, n_estimators=100, random_state=52
SG	max_iter = 1100, tol=1e−3

X_train, X_test, y_train, y_test = train_test_split(X, y, test_size =0.3, random_state =52, stratify =y)

where the parameter, ‘stratify’ preserves the proportion of target samples in the train and test datasets in the same ratio as the original dataset.

### Proposed methodology

**Figure 1:**
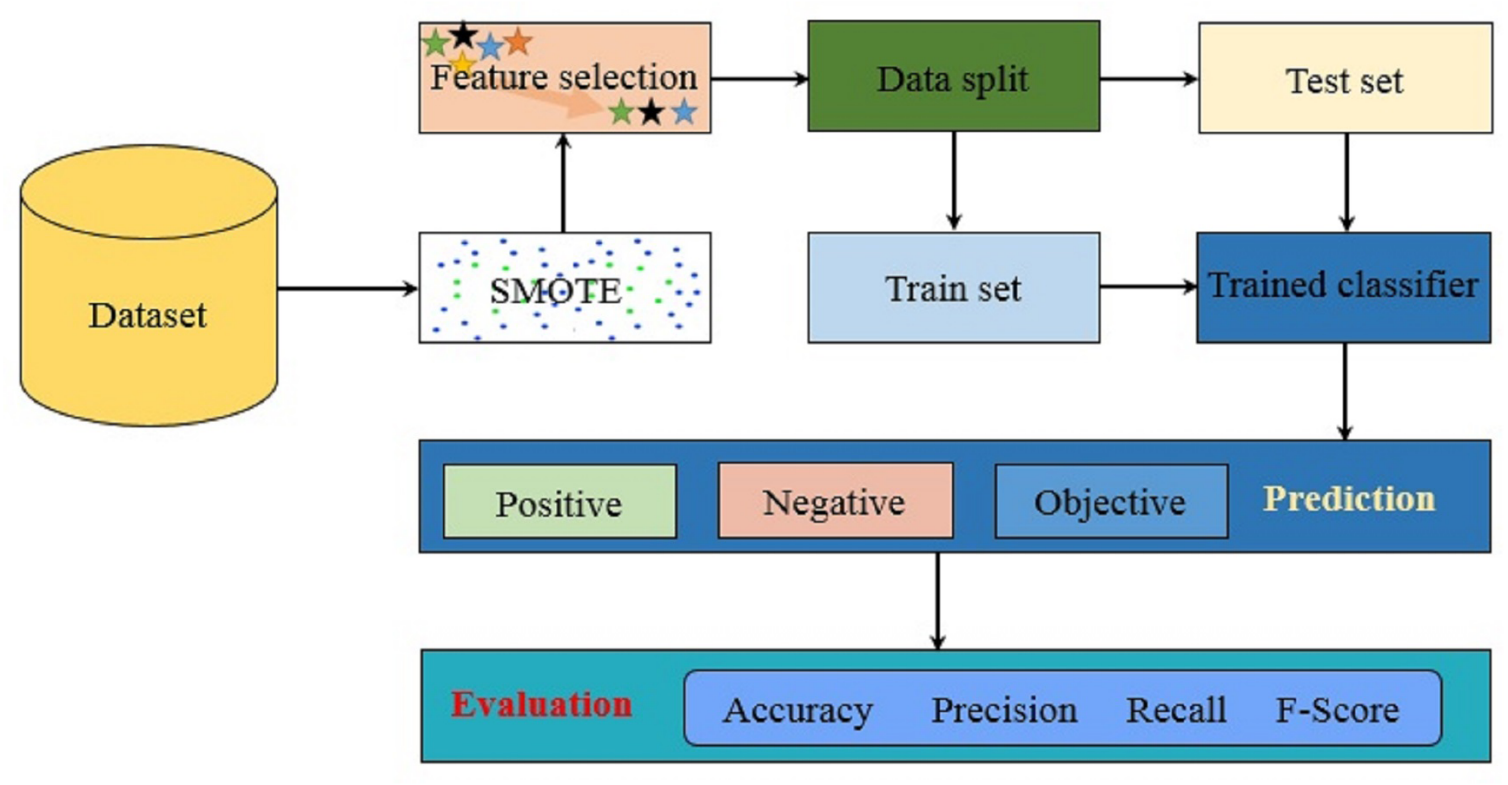
The architecture of the proposed methodology.

The proposed methodology aims at achieving high accuracy for sentiments of a citation and leverages various machine learning algorithms trained on the corpus. For this purpose, several courses of action have been adopted to evaluate the method with high accuracy. Figure 1 shows the architecture of the proposed methodology. Starting with the dataset acquisition, the synthetic minority oversampling technique SMOTE is applied to balance the dataset. Afterward, feature selection is applied where uni-gram and bi-gram TF-IDF are extracted. The data is split into training and testing subsets for training and testing, respectively. Trained models are later used for the prediction of sentiments for the unseen data. Experiments are performed with a three-fold purpose. First, the impact of data imbalance is evaluated where the experiments are performed with and without sampling technique. The SMOTE is adopted to perform the upsampling of the data samples for the minor classes to equal the number of samples to that of the major class.

**Figure 2:**
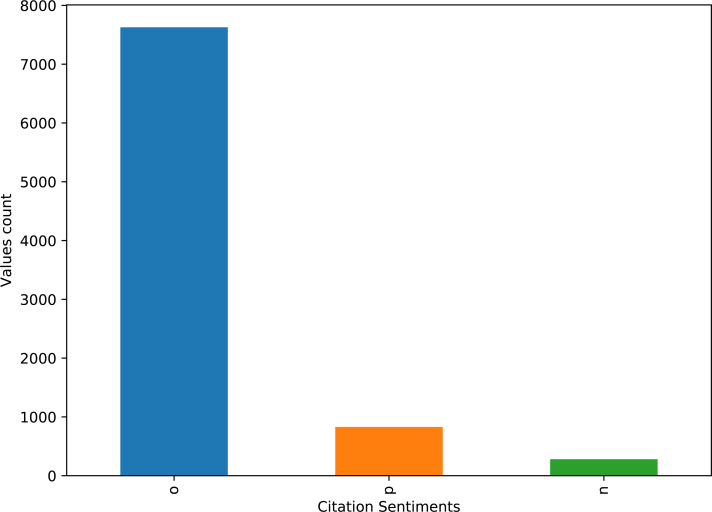
Distribution of the total number of records for positive, negative, and neutral classes in the dataset.

The dataset contains highly imbalanced samples for positive, negative, and objective classes. The distribution of the number of records for each class is given in Fig. 2.

SMOTE is one of the most commonly used sampling approaches which can be used to upsample the records for the minor class to elevate the performance of machine learning classifiers. For the current study, K nearest neighbor is used to produce the samples for the minor class. Instead of mere replacement, the minority classes are oversampled by creating synthetic instances. The aim of utilizing the SMOTE oversampling is to reduce the bias during the training process as the machine learning algorithms have major class biasness which increases the number of wrong predictions. SMOTE is selected on its reported performance which is better than other oversampling approaches like cluster centroid and adaptive synthetic (Chawla et al., 2002)

After SMOTE is applied to balance the dataset, feature extraction is performed to train the selected classifiers. This study leverages the TF-IDF features to this end. TF-IDF is a popular and widely used feature extraction technique for text analysis. Text analysis includes two important tasks of indexing and weighting and TF-IDF is considered for finding the weight of each term in a given document (Zhang, Yoshida & Tang, 2011). TF-IDF is the product of TF which determines the frequency of a term and IDF determines rare tokens in a given dataset. The mathematical forms of TF and IDF are as follows

(6) \begin{eqnarray*}TF(t)= \frac{N}{D} \end{eqnarray*}

(7) \begin{eqnarray*}IDF(t)=log \frac{d}{dt} \end{eqnarray*}

where *N* shows the occurrence of term *t*, *D* is the total number of terms in a given document, and *d* and *dt* represent the total number of documents and number of documents wherein term *t* appears. TF-IDF can be calculated as

(8) \begin{eqnarray*}{W}_{t,d}=T{F}_{t,d} \left( \frac{N}{{D}_{f,t}} \right) .\end{eqnarray*}

**Figure 3:**
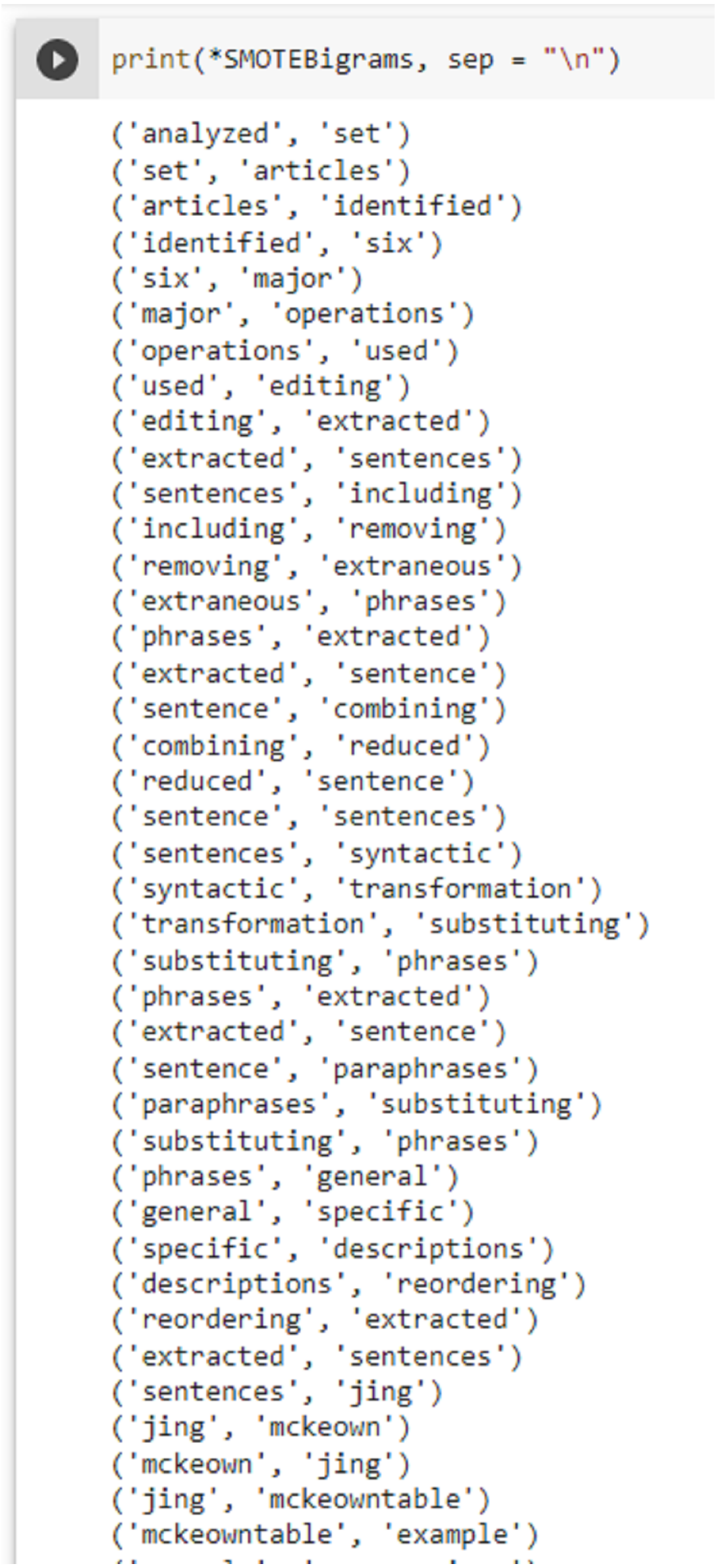
SMOTE bi-gram samples from the dataset used in this study.

This study uses both uni-grams and bi-grams for training the models to analyze their suitability for citation sentiment classification. A few SMOTE-based sample bi-grams are shown in Fig. 3.

Once the model is trained on the extracted features for the training set, it is used to make predictions for three classes of the dataset using the test set. The performance of the proposed methodology is evaluated using several well-known performance evaluation parameters.

**Accuracy** is one of the most widely used measures to evaluate the performance of trained models. Its values vary between 0 and 1 where the value closer to 1 indicates higher accuracy of a model. The following formula is used for accuracy

(9) \begin{eqnarray*}Accuracy= \frac{TP+TN}{TP+FP+TN+FN} \end{eqnarray*}

where the definition for TP, TN, FP, and FN is given as

**True Positive (TP)** when the sentiment of a citation is prediction positive and the actual label is also positive.

**True Negative (TN)** when the models predict the citation sentiment as negative and the actual label is also negative.

**False Positive (FP)** when the predicted sentiment is positive but the actual label is negative.

**False Negative (FN)** when the actual label is positive but the model predicts it to be negative.

Besides the accuracy, precision and recall are used as well to indicate the models’ performances with the following equations

(10) \begin{eqnarray*}Precision= \frac{TP}{TP+FP} \end{eqnarray*}

(11) \begin{eqnarray*}Recall= \frac{TP}{TP+FN} .\end{eqnarray*}

Also, F-score is used as well because it is more appropriate than merely using precision and recall. It shows a balance between the precision and recall by considering both and calculating their harmonic mean using

(12) \begin{eqnarray*}F-score=2\times \frac{Precision\times Recall}{Precision+Recall} .\end{eqnarray*}

## Results and Discussions

Experiments are performed with and without SMOTE for the proposed methodology to classify the sentiment of the citations. Similarly, the TF-IDF is used with uni-gram and bi-gram and the results are discussed separately.

### Performance of classifiers with imbalanced dataset

Initially, the classifiers are tested with the imbalanced dataset without using the SMOTE approach, both with uni-gram and bi-gram TF-IDF. The data split ratio is the same for all the experiments, *i.e.,* 70:30 for training and testing, respectively. Results with imbalance dataset and uni-gram features are given in Table 4.

**Table 4:** Machine learning classifiers’ results with imbalanced classes dataset and uni-gram features.

Classifier	Accuracy	Precision	Recall	F-Score
DT	0.8473	0.84	0.85	0.84
AB	0.8752	0.85	0.88	0.85
LR	0.8714	0.84	0.87	0.82
SG	0.8870	0.87	0.89	0.86
RF	0.8760	0.84	0.88	0.84
VC (LR+SG)	0.8725	0.86	0.87	0.82
ET	0.8775	0.85	0.88	0.84
SV	0.8961	0.87	0.89	0.87

Results show that the SV classifier outperforms all other classifiers concerning the accuracy, and F-score while sharing the same performance regarding precision and recall with the SG classifier. The highest accuracy is 0.8961 by SV followed by SG with an accuracy of 0.8870. The DT has the lowest accuracy on the imbalanced dataset and its precision and recall are also the lowest. On the other hand, the lowest F-score is for the LR and voting classifier. Generally speaking, the performance of all the classifiers is appropriate except for the DT if the class imbalance is considered. For the influence of bi-gram TF-IDF features, Table 5 can be observed.

**Table 5:** Performance of learning classifiers’ with imbalanced dataset and bi-gram features.

Classifier	Accuracy	Precision	Recall	F-Score
DT	0.8267	0.82	0.83	0.82
AB	0.8653	0.83	0.87	0.83
LR	0.8687	0.86	0.87	0.81
SG	0.8828	0.86	0.88	0.86
RF	0.8760	0.85	0.88	0.85
VC (LR+SG)	0.8679	0.87	0.87	0.81
ET	0.8701	0.85	0.88	0.85
SV	0.8828	0.86	0.88	0.86

The performance of the classifiers is slightly affected when bi-gram features are used. For example, the classification accuracy of DT, AB, LR, SG, VC, ET, and SV is reduced while RF performs similarly with both the uni-gram and bi-gram. The performance with bi-gram is usually reduced due to the rare occurrence of n-gram tokens in the text. The models assign higher weights to the rare tokes and IDF increases which affects the performance of the models.

### Classification results with balanced dataset without SMOTE

Dataset is balanced without SMOTE as well by considering an equal number of samples from each class. We can say that it is undersampling where the number of samples in the minor class is counted and a similar number of samples are taken from one or multiple major classes. For the selected dataset, the number of samples for the minor class is 280; therefore, 280 records are randomly selected from the other two classes as well. Using this subsampled dataset experiments are performed and the results are shown in Table 6.

**Table 6:** Machine learning classifiers’ results with the balanced dataset by selecting 280 samples from each class and unigram features.

Classifier	Accuracy	Precision	Recall	F-Score
DT	0.6904	0.69	0.69	0.69
AB	0.6547	0.66	0.65	0.66
LR	0.7539	0.75	0.75	0.75
SG	0.7698	0.77	0.77	0.77
RF	0.7500	0.75	0.75	0.75
VC (LR+SG)	0.7341	0.74	0.73	0.73
ET	0.7778	0.78	0.78	0.77
SV	0.7500	0.75	0.75	0.75

Table 6 shows the results using the unigram TF-IDF features on 280 records from each class. Results indicate that the performance of each classifier is degraded substantially. The highest accuracy is achieved by ET, *i.e.,* 0.7778 while the DT performs the worst with an accuracy of 0.6904. The models become overfit for the smaller datasets which leads to poor performance except for a few linear classifiers that can perform better with smaller datasets. To analyze the impact of bigram TF-IDF features, separate experiments are performed and the results are given in Table 7.

**Table 7:** Results using bi-gram features with the balanced dataset by selecting 280 samples from each class.

Classifier	Accuracy	Precision	Recall	F-Score
DT	0.6904	0.68	0.69	0.69
AB	0.6111	0.64	0.61	0.60
LR	0.7461	0.75	0.75	0.74
SG	0.7658	0.77	0.77	0.76
RF	0.7261	0.73	0.73	0.72
VC (LR+SG)	0.7539	0.75	0.75	0.75
ET	0.7301	0.76	0.73	0.72
SV	0.7698	0.78	0.77	0.77

Results using bi-gram TF-IDF features suggest that the performance of the classifier is enhanced than that of using the uni-gram features. Commonly bi-gram features may perform worse than the uni-grams especially when extra features are added which is not the case in the current study. Traditionally, longer n-grams are rare and lead to higher IDF values which affect the results. Using the bi-gram features, the performance of AB, LR, SG, RF, and ET has degraded while VC and SV experience a boost in performance while DT has no change.

### Performance of classifiers using SMOTE

The dataset is balanced using SMOTE where the samples from the minor class are oversampled to equal their samples to that of the major class. Experiments are performed on the balanced dataset and results are shown in Table 8 for uni-gram features. Results indicate that the performance of all the classifiers has been elevated substantially using the SMOTE balanced dataset than both of imbalanced and sub-sampled balanced datasets. All classifiers show an accuracy of higher than 0.90 except for the AB classifier while RF, ET, and SV show accuracy scores of higher than 0.96.

**Table 8:** Results for classifiers with balanced dataset using SMOTE and uni-gram features.

Classifier	Accuracy	Precision	Recall	F-Score
DT	0.9010	0.90	0.90	0.90
AB	0.8361	0.84	0.79	0.82
LR	0.9388	0.94	0.94	0.94
SG	0.9361	0.96	0.96	0.96
RF	0.9729	0.98	0.96	0.97
VC (LR+SG)	0.9418	0.94	0.94	0.94
ET	0.9826	0.98	0.98	0.98
SV	0.9669	0.97	0.97	0.97

The performance of the classifiers is increased with the upsampling because it increases the statistical significance of the models. SMOTE generates synthetic samples that are more representative of the training corpus which helps in the training process and increases the classification accuracy. For bi-gram features results as shown in Table 9, the performance is increased further than that of using the uni-gram features. Bi-grams features did not perform well on the imbalanced and sub-sampled balanced dataset due to the smaller number of training samples. Longer n-grams tend to be rare in the text which leads to higher IDF weights which leads to the poor performance of the classifiers in most cases. However, when the training corpus contains a large number of samples, the probability of finding n-grams is high. Since n-grams are not rare in a large corpus, lower IDF weights are assigned and the performance is improved.

**Table 9:** Classification results with balanced dataset using SMOTE and bi-gram features.

Classifier	Accuracy	Precision	Recall	F-Score
DT	0.9254	0.93	0.93	0.93
AB	0.7969	0.81	0.80	0.80
LR	0.9765	0.98	0.98	0.98
SG	0.9896	0.99	0.99	0.99
RF	0.9766	0.98	0.98	0.98
VC (LR+SG)	0.9800	0.98	0.98	0.98
ET	0.9857	0.99	0.99	0.99
SV	0.9900	0.99	0.99	0.99

### Performance comparison with state-of-the-art approaches

For evaluating the performance of the proposed methodology, its accuracy is compared with several state-of-the-art approaches. These approaches utilize several machine learning algorithms with different features from the citation corpus to achieve high accuracy. Table 10 shows the model and features that achieved the highest accuracy as reported in these papers. Results show that the proposed approach outperforms the state-of-the-art techniques. Tuning of various hyperparameters and the SMOTE oversampling helps to achieve higher accuracy than those of other approaches. Extensive experiments with imbalanced, down-sampled balanced and SMOTE balanced datasets help to identify the problems associated with these approaches, and consequently a better methodology is improvised to achieve high citation sentiment accuracy.

**Table 10:** Performance comparison of the proposed methodology with state-of-the-art approaches.

Reference	Model	Features	Accuracy
Ghosh & Shah (2020)	Dagging	Sentence score, +ve n-grams, -ve n-grams, part-of-speech, dependency tags, self-citation, sentiment words	80.61%
Ikram & Afzal (2019)	SVM	Citation aspects, n-grams (2,3,5)	81.90%
Mercier et al. (2020)	CNN	–	88.93%
Proposed	SVM	bi-gram (SMOTE upsampled)	99.0%

## Discussions

Citations serve as an important indicator for scientific research articles and are further used to calculate performance-related parameters like i-index, h-index, etc. However, citations are used as the objective measure where their frequency is used for h-index and similar parameters; the subjective aspect is ignored in this regard. Since all citations do not appreciate a scientific work and are also used to criticize a research article, considering their context would provide better insights into the quality of an article. In this regard, this performs classification on the sentiment in which a citation is used. Classifying the citation sentiments into positive, negative, and neutral can provide complement the objective measure of merely counting the citation thus showing a more realistic picture of an article’s quality. In this regard, experiments are performed using an imbalanced dataset and balanced datasets using undersampling and oversampling on a textual dataset that contains the cited text from scientific research articles. In addition, the influence of using uni-gram and bi-gram is also investigated. Experimental results indicate that the highest accuracy can be obtained using bi-gram TF-IDF features from the upsampled dataset. Using upsampling, models have enough data to get a good fit and show better performance than the under-sampled dataset. SV shows a 99.0% accuracy with bi-gram features while ET shows a 98.26% accuracy with uni-gram features. These results are better than existing methods.

### Threats to validity

This study performs both under-sampling and over-sampling for experiments where each has its own threats to validity. Since under-sampling randomly removes the samples from the majority class to balance the minority class samples, information loss can lead to model underfitting. Similarly, when the imbalance between the minor and major class samples is large, class clusters may invade the space of each other thus leading to model overfitting. The impact of the level of imbalance between classes is yet to be investigated. In addition, the best results are obtained using uni-gram and bi-gram TF-IDF features with ET and SV classifiers, respectively. For obtaining the best performance, several hyperparameters are optimized regarding the dataset used for experiments. It should be made clear that changing the models or using a different set of hyperparameters may yield very different results. The same is true for using a different feature extraction approach.

## Conclusion

This study proposes a novel methodology to classify the sentiment of citations for the comprehension of the quality and importance of scientific articles. Experiments are carried out with a three-fold purpose: accuracy with the imbalanced dataset, the influence of down-sampled balanced and oversampled balanced dataset, and the impact of uni-gram and bi-gram TF-IDF features. The imbalanced dataset shows poor results for all the classifiers with the highest accuracy of 89.61% and 88.28% from SV and SG using uni-gram and bi-gram features, respectively. For a down-sampled balanced dataset, an equal number of samples from all three classes are randomly selected. Experiment results indicate that the performance is degraded and the highest accuracy is reduced to 77.78 for ET and 76.98% for SV with uni-gram and bi-gram features, respectively. Further experiments using SMOTE oversampling show that performance has been improved substantially with both the features. The highest accuracy of 98.26% is achieved with the RF using the uni-gram features while for bi-gram features the highest accuracy of 99.0% is reached with the SV classifier. Performance comparison with the state-of-the-art approaches suggests that the proposed approach is far better than other approaches for accurately classifying the sentiment of citations. The current study only considers the citation sentiment for scientific articles, however, the impact of citation sentiment on a research article’s importance is not determined. In the future, we intend to use the citation sentiment score to adjust the h-index for scientific articles, as well as, authors. Currently, the h-index is calculated using the simple count of citations and does not consider the citation sentiment.

## Supplemental Information

10.7717/peerj-cs.1107/supp-1 Supplemental Information 1 The dataset and all codes used for experiment

## Additional Information and Declarations

### Data Availability

The following information was supplied regarding data availability: The code and data are available at the University of Cambridge:
https://cl.awaisathar.com/citation-sentiment-corpus/.
