# Supplementary material for: Comprehension of polarity of articles by citation sentiment analysis using TF-IDF and ML classifiers
Source: PeerJ Comput Sci. 2022 Dec 13;8:e1107. doi: 10.7717/peerj-cs.1107 (PMC10280177; doi:10.7717/peerj-cs.1107)
Supplement: Supplemental Information 1 [file peerj-cs-08-1107-s001.zip › Experimental Results.docx]

**Original dataset statistics:**

Neutral 7627

positive 829

negative 280

**Features:**

**TF/IDF (unigram, unigram+bi-gram Fusion)**

**data.columns = ["Source_Paper_ID", "Target_Paper_ID", "Sentiment", "Citation_Text"]**

**Dataset Link:**

[**https://cl.awaisathar.com/citation-sentiment-corpus/**](https://cl.awaisathar.com/citation-sentiment-corpus/)

Table 1 Machine Learning Classifier Results with imbalanced classes

| **Models** | **Accuracy** | **Precision** | **Recall** | **F-Score** |
| --- | --- | --- | --- | --- |
| Decision Tree | 0.8473 | 0.84 | 0.85 | 0.84 |
| AdaBoost Classifier | 0.8752 | 0.85 | 0.88 | 0.85 |
| Logistic Regression | 0.8714 | 0.84 | 0.87 | 0.82 |
| Stochastic Gradient Classifier | 0.8870 | 0.87 | 0.89 | 0.86 |
| Random Forest Classifier | 0.8760 | 0.84 | 0.88 | 0.84 |
| Voting Classifier LR+SGD | 0.8725 | 0.86 | 0.87 | 0.82 |
| Extra Tree Classifier | 0.8775 | 0.85 | 0.88 | 0.84 |
| Support Vector Classifier | 0.8961 | 0.87 | 0.89 | 0.87 |

Table 2 Machine Learning Classifier Results with **balanced** classes (280 records of each class)

| **Models** | **Accuracy** | **Precision** | **Recall** | **F-Score** |
| --- | --- | --- | --- | --- |
| Decision Tree | 0.6904 | 0.69 | 0.69 | 0.69 |
| AdaBoost Classifier | 0.6547 | 0.66 | 0.65 | 0.66 |
| Logistic Regression | 0.7539 | 0.75 | 0.75 | 0.75 |
| Stochastic Gradient Classifier | 0.7698 | 0.77 | 0.77 | 0.77 |
| Random Forest Classifier | 0.7500 | 0.75 | 0.75 | 0.75 |
| Voting Classifier LR+SGD | 0.7341 | 0.74 | 0.73 | 0.73 |
| Extra Tree Classifier | 0.7777 | 0.78 | 0.78 | 0.77 |
| Support Vector Classifier | 0.7500 | 0.75 | 0.75 | 0.75 |

Table 3 Machine Learning Classifier Results with **SMOTE up sampling**

| **Models** | **Accuracy** | **Precision** | **Recall** | **F-Score** |
| --- | --- | --- | --- | --- |
| Decision Tree | 0.9010 | 0.90 | 0.90 | 0.90 |
| AdaBoost Classifier | 0.8361 | 0.84 | 0.79 | 0.82 |
| Logistic Regression | 0.9388 | 0.94 | 0.94 | 0.94 |
| Stochastic Gradient Classifier | 0.9361 | 0.96 | 0.96 | 0.96 |
| Random Forest Classifier | 0.9729 | 0.98 | 0.96 | 0.97 |
| Voting Classifier LR+SGD | 0.9418 | 0.94 | 0.94 | 0.94 |
| Extra Tree Classifier | 0.9826 | 0.98 | 0.98 | 0.98 |
| Support Vector Classifier | 0.9669 | 0.97 | 0.97 | 0.97 |

Bi-gram imbalanced

| **Models** | **Accuracy** | **Precision** | **Recall** | **F-score** |
| --- | --- | --- | --- | --- |
| Decision Tree | 0.8267 | 0.82 | 0.83 | 0.82 |
| AdaBoost Classifier | 0.8653 | 0.83 | 0.87 | 0.83 |
| Logistic Regression | 0.8687 | 0.86 | 0.87 | 0.81 |
| Stochastic Gradient Classifier | 0.8828 | 0.86 | 0.88 | 0.86 |
| Random Forest Classifier | 0.8760 | 0.85 | 0.88 | 0.85 |
| Voting Classifier LR+SGD | 0.8679 | 0.87 | 0.87 | 0.81 |
| Extra Tree Classifier | 0.8701 | 0.85 | 0.88 | 0.85 |
| Support Vector Classifier | 0.8828 | 0.86 | 0.88 | 0.86 |

Bi-gram balanced

| **Models** | **Accuracy** | **Precision** | **Recall** | **F-score** |
| --- | --- | --- | --- | --- |
| Decision Tree | 0.6904 | 0.68 | 0.69 | 0.69 |
| AdaBoost Classifier | 0.6111 | 0.64 | 0.61 | 0.60 |
| Logistic Regression | 0.7461 | 0.75 | 0.75 | 0.74 |
| Stochastic Gradient Classifier | 0.7658 | 0.77 | 0.77 | 0.76 |
| Random Forest Classifier | 0.7261 | 0.73 | 0.73 | 0.72 |
| Voting Classifier LR+SGD | 0.7539 | 0.75 | 0.75 | 0.75 |
| Extra Tree Classifier | 0.7301 | 0.76 | 0.73 | 0.72 |
| Support Vector Classifier | 0.7698 | 0.78 | 0.77 | 0.77 |

Bi-gram smote

| **Models** | **Accuracy** | **Precision** | **Recall** | **F-score** |
| --- | --- | --- | --- | --- |
| Decision Tree | 0.9254 | 0.93 | 0.93 | 0.93 |
| AdaBoost Classifier | 0.7969 | 0.81 | 0.80 | 0.80 |
| Logistic Regression | 0.9765 | 0.98 | 0.98 | 0.98 |
| Stochastic Gradient Classifier | 0.9896 | 0.99 | 0.99 | 0.99 |
| Random Forest Classifier | 0.9766 | 0.98 | 0.98 | 0.98 |
| Voting Classifier LR+SGD | 0.9800 | 0.98 | 0.98 | 0.98 |
| Extra Tree Classifier | 0.9857 | 0.99 | 0.99 | 0.99 |
| Support Vector Classifier | 0.9900 | 0.99 | 0.99 | 0.99 |
